# Car Observations

**Published:** 1859-10

**Authors:** 


### CAR OBSERVATIONS.

The city cars are an institution, that is to say. a social necessity. We seldom enter one of them without finding it crowded with—human nature.

One man enters, and, being too warm, at once hoists a window, without the first thought of consulting the wishes of the person next him; every one seems to think that the window nearest himself is wholly his own personal property for the time being. Another prefers to stand erect in the forward door, thus monopolising for himself the breeze which properly belongs to the whole company.

But the car is full, full it may be to an almost suffocating point, or, if an omnibus, all the seats are taken, when, if the vehicle is hailed for another passenger, male or female, there scarcely ever fails to be a growl from some quarter, in a hoarse surly tone, "full, driver." In almost all cases it will be found that these uncouth objurgations come from youths about twenty. It is a very rare thing to hear any objection coming from an old man, or from a married man. And why should any one object who has a good and full seat for himself? The person wanting to enter may barely have time to reach the last train for the day and to "lay over" may be pecuniary ruin; may be to miss a friend or relative, a child, a wife, a sister going on a long, mayhap a returnless journey; it may be a messenger for a physician, or from a physician, bearing most important remedies, or counsel of vital interest; or a rain may be threatened which endangers a "best suit," purchased by the labour and the savings of weary months; for the bare risk of emergencies like these, shame to the ugly churl, who, himself supplied, would refuse accommodation to a fellow-citizen.

On a beautiful September morning, the Fourth Avenue downward car was full of men, several were standing, when the bell tingled a halt, and a woman entered, dressed plainly, in the deepest black, without gloves, holding in her hand a small package in coarse straw paper; her countenance was the personification of a deep long grief. As we were one of the farthest from her, our eye ran along the two lines to see if there was any one among the crowd who would be likely to offer a seat; we felt certain of but one in the thirty, most of whom seemed to be clerks, book-keepers, or men who were well to do. The person selected was not a handsome man in any one feature, but the whole *contour* of countenance and eyes gave the impression that he was a gentleman; there was a frank and manly face, a quickness of eye, and a personal neatness, with a general expression of good-will, which made us sure of our mark; all this time—half a minute—the pale, sorrowing face made its way nearer and nearer, but there was not the slightest show of offering a seat; our favorite kept his eye steadily on the saddened features, and the moment their eyes met, his seat was vacated, with a kindly intimation that it was at the service of the new comer. In a very short time he obtained a vacated seat, so he lost nothing and saved his politeness. After he left we inquired his name, and J. M. Nixon, of the house of Doremus and Nixon will excuse this use of his name, for we shall never meet him again without feeling that there is a man, for he respected not youth or beauty, or wealth, or station, for there were none of these in the unpretending passenger, but he respected himself and all of womankind.

